# Concordance of assessments of four PD-L1 immunohistochemical assays in esophageal squamous cell carcinoma (ESCC)

**DOI:** 10.1007/s00432-023-05595-0

**Published:** 2024-01-28

**Authors:** Xinran Wang, Jiankun He, Jinze Li, Chun Wu, Meng Yue, Shuyao Niu, Ying Jia, Zhanli Jia, Lijing Cai, Yueping Liu

**Affiliations:** Department of Pathology, The Fourth Hospital of Hebei Medical University, No. 12 Jiankang Road, Shijiazhuang, 050011 Hebei China

**Keywords:** PD-L1, Scoring methods, Concordance, ESCC

## Abstract

**Objective:**

Given real-world limitations in programmed death-ligand 1 (PD-L1) testing, concordance studies between PD-L1 assays are needed. We undertook comparisons of PD-L1 assays (DAKO22C3, Ventana SP263, Ventana SP142, E1L3N) among observers in esophageal squamous cell carcinoma (ESCC) to provide information on the analytical and clinical comparability of four PD-L1 IHC assays.

**Methods:**

Paraffin embedded samples of 50 cases of esophageal squamous cell carcinoma were obtained, satined with all four PD-L1 assays. PD-L1 was evaluated by 68 pathologists from 19 different hospitals. PD-L1 expression was assessed for combined positive score (CPS).

**Results:**

The expression sensitivity of SP263 was the highest in ESCC, followed by 22C3, E1L3N and SP142. Taking CPS 10 as the critical value, inter-observer concordance for CPS scores among 68 physicians was assessed for the 22C3, SP263, SP142, and E1L3N assays, yielding values of 0.777, 0.790, 0.758, and 0.782, respectively. In the comparison between assays, the overall CPS scores concordance rates between 22C3 and SP263, SP142, and E1L3N were 0.896, 0.833, and 0.853, respectively. 22C3 and SP263 have high concordance, with OPA of 0.896, while E1L3N and SP142 have the highest concordance, with OPA of 0.908.

**Conclusion:**

In ESCC, the concordance of PD-L1 evaluation among observers is good, and the immune cell score is still an important factor affecting the concordance of interpretation among observers. Cases near the specific threshold are still the difficult problem of interpretation. SP263 had the highest CPS score of the four assays. SP263 cannot identify all 22C3 positive cases, but had good concordance with 22C3.E1L3N and SP142 showed high concordance.

**Supplementary Information:**

The online version contains supplementary material available at 10.1007/s00432-023-05595-0.

## Introduction

Esophageal cancer is the most common malignant tumor of the digestive tract. Globally, it is responsible for approximately 300,000 deaths yearly. China has one of the highest incidence rates of esophageal cancer in the world, about 90% of which are squamous cell carcinomas (Song et al. 2014). Esophageal squamous cell carcinoma (ESCC) has poor prognosis, is highly aggressive and has an overall 5-year survival rate of less than 15% (Talukdar et al. 2018). Programmed cell death protein 1 (PD-1) and programmed cell death ligand 1 (PD-L1) on the tumor cells binds to programmed cell death ligand 2 (PD-L2), and thereby confers immune evasion abilities to the tumor cells. With the development of PD-1/PD-L1 immunotherapy, esophageal squamous cell carcinoma has ushered in a new era for immunotherapy.

The expression of PD-L1 protein in surviving cancer cells and immune cells, as determined by immunohistochemistry (IHC), correlates with the therapeutic effects of immune checkpoint inhibitors. Results from the KEYNOTE-181 trial (Kojima et al. 2020) showed that in patients with recurrent or metastatic ESCC with PD-L1 (CPS ≥ 10), compared with chemotherapy, pembrolizumab monotherapy prolonged overall survival (median OS: 9.3 months vs. 6.7 months) and reduced the risk of death by 36% (HR = 0.64 95% CI 0.46–0.90). Based on the results of the KEYNOTE-181 trial [NCT02564263], in July 2019 the FDA approved pembrolizumab for the second-line treatment of patients with PD-L1-positive, locally advanced or metastatic esophageal squamous cell carcinoma and also approved PD-L1 Dako 22C3 pharmDx test as a companion diagnostic for pembrolizumab.

The reliability of PD-L1 interpretation is important for the selection of patients for immunotherapy. Since each PD-L1 IHC assay uses a different PD-L1 clone number and a different immunohistochemical staining platform, therefore, each PD-L1 antibody may possess its own staining characteristics. This poses a significant challenge for most pathology departments in performing PD-L1 assays. Previous studies (Sound et al. 2018; Keppens et al. 2020; Hirsch et al. 2017; Ricci et al. 2020) have evaluated the similarities and differences between PD-L1 IHCs of different clone numbers in non-small cell lung cancer (NSCLC). The Blueprint Project phase 2 (Sound et al. 2018) (2018) evaluated the concordance of five PD-L1 antibodies in 81 NSCLC samples. The results demonstrated a high concordance amongst the staining tests of 22C3, 28-8 and SP263. The sensitivity of SP142, however, was low and its concordance with the aforementioned three antibodies was poor. A few studies (Sound et al. 2018; Rakha et al. 2017; Reisenbichler et al. 2020; Wang et al. 2021) have examined the reproducibility, in NSCLC and breast cancer, of PD-L1 interpretation concordance between inter-department pathologists and intra-department pathologists. The results showed a good concordance between the ‘intra-department’ and the ‘inter-department’ groups of pathologists. To the best of our knowledge few study has been conducted on the concordance of PD-L1 expression in patients with esophageal squamous cell carcinoma. Moreover, the physicians involved in interpretation in other studies are rather few, and the interpretation factors affecting concordance have not been elucidated.

This study is the first to analyze and make a comparison of the concordance scores among pathologists for four PD-L1 antibodies in esophageal squamous cell carcinoma. In addition, 68 pathologists from 18 medical institutions were recruited to individually interpret four antibodies, namely, 22C3, SP263, SP142 and E1L3N, with the aim of investigating interobserver concordance of PD-L1 evaluation in esophageal squamous cell carcinoma, making comparisons between different antibodies, and further analyzing the factors affecting the concordance of interpretations.

## Materials and methods

### Case selection

Fifty paraffin-embedded samples were selected. All the patients were diagnosed with esophageal squamous cell carcinoma obtained from surgical resections performed at the Fourth Hospital of Hebei Medical University between December 2018 and December 2019. All patients did not receive neoadjuvant chemotherapy with clinical stage T2–T4. Tissues with carcinoma in situ and poor tissue fixation were removed. All tissues and data retrieval were approved by the Institutional Research Ethics Committee of the Fourth Hospital of Hebei Medical University on September 17, 2020 and completed with the application number 2020KY118. The study involved tissue materials of human participants. Informed consent and disclosure of identifiable patient information had been obtained when using existing pathological materials.

### PD-L1 immunohistochemical staining and section scanning

All specimens were fixed in 10% neutral buffered formalin fixative within 1 h of isolation. Fixation time was 6–72 h.Fifty samples were continuously sliced and at least five tissue sections were obtained per sample. HE staining and PD-L1 antibody staining were performed separately. Sufficient tumor tissues were identified on hematoxylin and eosin stained sections along with no less than 100 live tumor cells and their associated mesenchymal immune cells. These tissue sections were then stained for PD-L1. All sections stained with PD-L1 strictly following the manufacturer’s instructions on automatic immunohistochemistry. PD-L1 22C3 (Dako North America Inc, Carpinteria, CA) staining was performed using the Dako Autostainer Link48 platform; PD-L1 Ventana SP263 (Ventana Medical Systems, Tucson, USA), PD-L1 E1L3N (Aide Biomedical Technology Co., Ltd, Xiamen, China) and PD-L1 Ventana SP142 assay kit (Ventana Medical Systems, Tucson, USA) staining were performed using the Ventana Benchmark Ultra platform. All sections were scanned at 40X magnification on a UNIC digital pathology scanner (PRECICE 600 series), and their complete scanned images (WSI) were collected.

### Recruitment of pathologists and the PD-L1 interpretation scoring process

We organized a multi-institutional ring study for PD-L1 assays assessment in esophageal carcimoma, recruiting 68 board-certified pathologists from 19 provincial and municipal hospitals, with diversity in their experience. They all specialize in pathology and work in hospitals with a median of 14 years of experience (5–25 y). There were 6 chief pathologist, 12 deputy chief pathologists, 36 attending pathologists, and 14 resident pathologist, among whom 47 had received 22C3 training, of whom 44 had also trained for SP263 at the same time. 39 pathologist had received the SP142 training.25 pathologist had received above training of the three assays. All pathologists accepted the invitation voluntarily. To reduce the intraobserver and interobserver variability caused by the heterogeneity of the interpretation time, all interpretations were completed on the same day. All the physicians attended the PD-L1 (22C3) CPS interpretation training in the morning and passed the examination regardless of whether they have received prior training or not. In the afternoon of the same day, all physicians interpreted four PD-L1 antibodies at the same time. According to the CPS interpretation guidelines (DAKO 2019): CPS = number of PD-L1 stained cells of any intensity (tumor cells, lymphocytes, macrophages) ÷ total number of live tumor cells × 100. Control HE sections were available for each case sample, and all antibodies were labeled according to the PD-L1 (22C3) CPS interpretation. All 68 physicians, through an online section reading platform, simultaneously, separately and independently carried out a CPS 0–100 continuity score on the 50 cases of 22C3, 263, 142, E1L3N. Moreover, pathologists were asked to score CPS, meanwhile, they are also asked to score the stained immune cells, tumor cells separately by each case. As shown below, we recorded the positive tumor cells as Tumor Cell Positive Score (TCPS) and the positive lymphocytes and macrophages as Immune Cell Positive Score, (ICPS). The pathologists can freely pan and reduce the entire section from the equivalent of 1× to 40× magnification for WSI.

### Statistical methods

Analyses were conducted using R (version 4.0.4, Vienna, Austria). The overall concordance rate (OPA), negative concordance rate (NPA), and positive concordance rate (PPA) were used to analyze the concordance of PD-L1 scores among 68 doctors of the 4 antibodies (Guidance for Industry and FDA Staff Statistical Guidance on Reporting Results from Studies Evaluating Diagnostic Tests 2007).For interobserver reproducibility, pairwise combination of any two pathologists, we quantify the OPA using the proportion of tissue samples upon which all selected pathologists agree. Calculation of OPA for 50 cases of 68 pathologists results in 113,900 pairs [C^2^_68_ (the number of comparison pairs of each case) × 50 (the number of cases)]. For comparability of different assays, calculation of OPA for 50 cases of 68 pathologists results in 3400 pairs [68 (the number of pathologists) × 50 (the number of cases)]. For each pairwise comparison among pathologists, the first doctor as regarded as the non-reference gold standard and the second as the new test,, the results (total pairs, T) were counted as concordant pairs (CPs), including negative-negative (NN) CPs, positive-positive (PP) CPs, and discordant pairs(DCPs)。

## Results

### Interobserver reproducibility of the four PD-L1 Assay

**Fig. 1:**
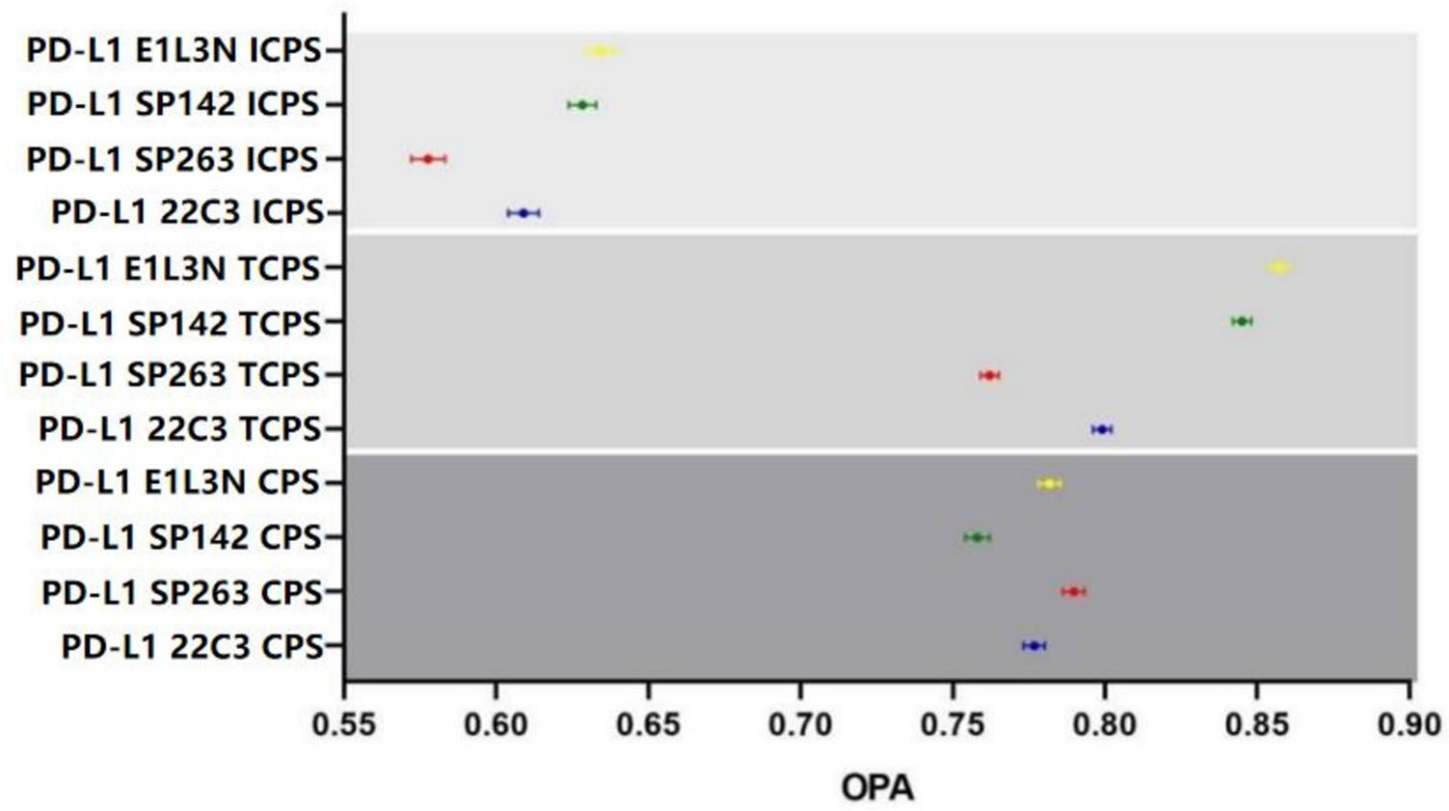
The overall concordance rate of CPS, TCPS and ICPs of four PD-L1 assays. The circles in the middle of the bars indicate the values, and the bars indicate a 95% confidence interval. The colors blue, red, green and yellow represent the 22C3, SP263, SP142 and E1L3N respectively. ICPS shows relatively poor concordance

Taking 10 as the positive threshold, the overall agreement rate (OPA) of the scores of the four antibodies 22C3, SP263, SP142, and E1L3N by 68 pathologists in 50 cases is shown in Supplementary Fig. 1. The overall concordance rates of CPS score of 22C3, SP263, SP142 and E1L3N were 0.777 (0.773–0.780), 0.790 (0.786–0.793), 0.758 (0.754–0.762) and 0.782 (0.778–0.785), respectively (Table 1). OPA results of CPS binary score of 68 pathologists for these four PD-L1 antibodies were shown in Fig. 1.

**Table 1:** Interobserver reproducibility of the four PD-L1 assays

Interobersever (N = 113,900)			
Measurements	CPS	TCPS	ICPS
22C3			
CPs	88,500 (77.7%)	91,006 (79.9%)	69,365 (60.9%)
DCPs	25,400 (22.3%)	22,894 (20.1%)	44,535 (39.1%)
Measures of agreement (95% CI)			
OPA	0.777 (0.773–0.780)	0.799 (0.796–0.802)	0.609 (0.604–0.614)
PPA	0.856 (0.851–0.860)	0.784 (0.779–0.790)	0.524 (0.514–0.535)
NPA	0.660 (0.652–0.669)	0.835 (0.831–0.839)	0.665 (0.658–0.672)
SP263			
CPs	89,981 (79.0%)	86,792 (76.2%)	65,834 (57.8%)
DCPs	23,919 (21.0%)	27,108 (23.8%)	48,066 (42.2%)
Measures of agreement (95% CI)			
OPA	0.790 (0.786–0.793)	0.762 (0.759–0.765)	0.578 (0.572–0.583)
PPA	0.878 (0.873–0.883)	0.790 (0.784–0.795)	0.561 (0.551–0.572)
NPA	0.554 (0.543–0.566)	0.763 (0.757–0.769)	0.605 (0.597–0.613)
SP142			
CPs	89,412 (75.8%)	96,246 (84.5%)	71,529 (62.8%)
DCPs	24,488 (24.2%)	17,654 (15.5%)	42,371 (37.2%)
Measures of agreement (95% CI)			
OPA	0.758 (0.754–0.762)	0.845 (0.842–0.848)	0.628 (0.624–0.633)
PPA	0.817 (0.811–0.823)	0.760 (0.755–0.766)	0.577 (0.567–0.586)
NPA	0.728 (0.721–0.736)	0.904 (0.902–0.907)	0.689 (0.682–0.696)
E1L3N			
CPs	93,398 (78.2%)	97,612 (85.7%)	72,213 (63.4%)
DCPs	20,502 (21.8%)	16,288 (14.3%)	41,687 (36.6%)
Measures of agreement (95% CI)			
OPA	0.782 (0.778–0.785)	0.857 (0.854–0.860)	0.634 (0.630–0.639)
PPA	0.839 (0.834–0.844)	0.795 (0.790–0.801)	0.534 (0.524–0.544)
NPA	0.745 (0.738–0.751)	0.907 (0.904–0.909)	0.701 (0.695–0.708)

N = C^2^_68_ (the number of comparison pairs of each case) × 50 (the number of cases)

*CI* confidence interval, *CP* concordant pair, *DCP* discordant CP, *NPA* negative percentage agreement, *OPA* overall percentage agreement, *PPA* positive percentage agreement

Among the four antibodies, the overall agreement rate of the CPS score of SP263 was higher than that of TCPS and ICPS, and the overall agreement rate of TCPS score of 22C3, SP142, and E1L3N was higher than that of CPS and ICPS. However, the overall agreement rate of the ICPS scores of the four antibodies was the worst (Fig. 1). The evaluation of immune cells may be the reason for the decreased concordance of pathologists’ scores. In addition, using 10 as the positive threshold, of the 22C3 CPS scores of the 50 cases we studied, 33 cases had inconsistent scores by pathologists, of which 29 cases had CPS scores between 5 and 20 and 4 cases were between 21 and 30. Among the SP263 CPS scores, 30 cases are inconsistent, 26 cases have CPS scores between 5 and 20, and 4 cases are between 21 and 28. In SP142 and E1L3N CPS scores, 34 and 33 cases are inconsistent, respectively. Among them, 28 cases and 31 cases are between 5 and 20. These cases with inconsistent scores were clustered near the positive threshold of 10, that is, between 5 and 20, and these cases near the threshold also contributed to the decrease in the overall consensus rate among pathologists.

### Comparability of PD-L1 staining between four assays

**Fig. 2:**
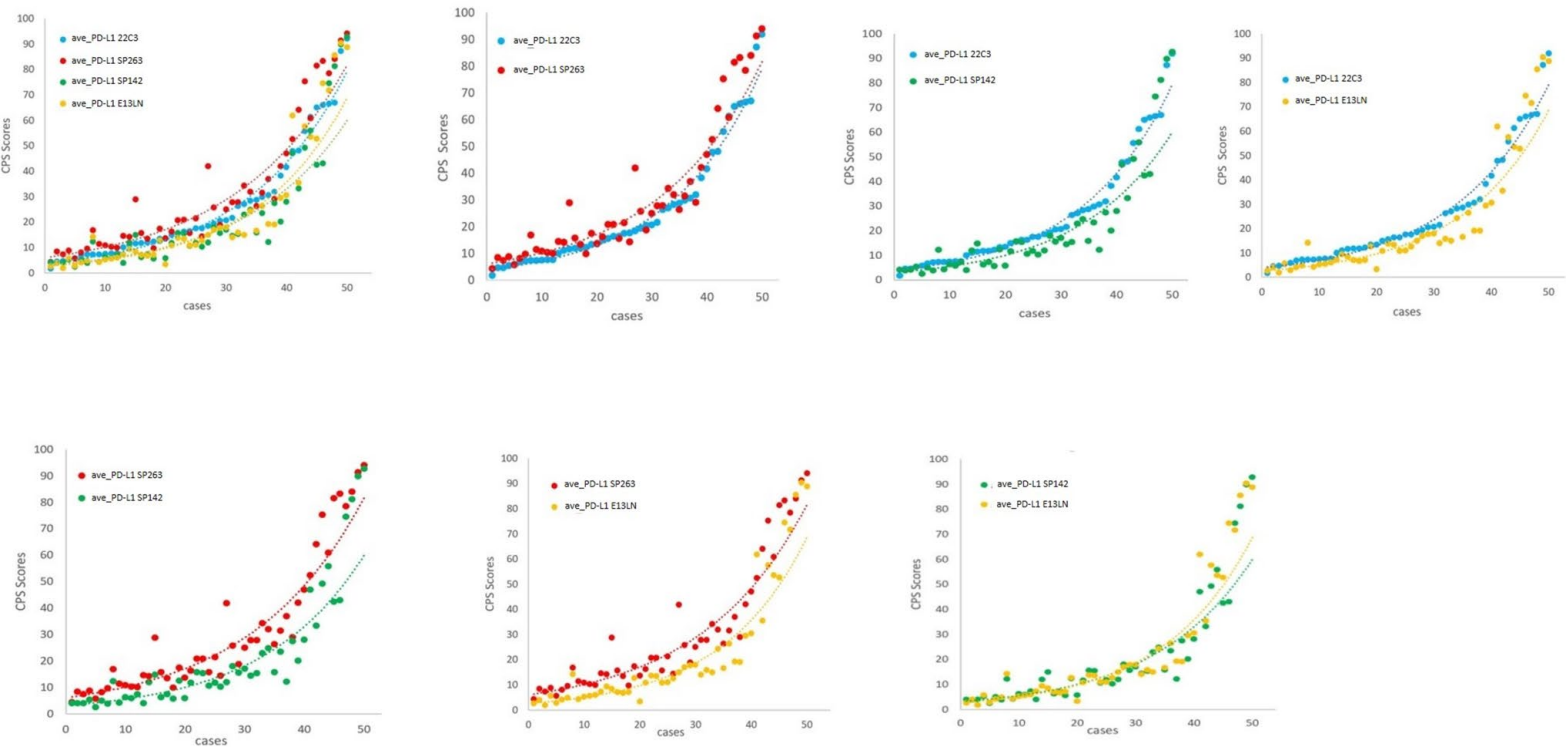
Comparison of CPS scores of four PD-L1 assays. Overall comparison (**A**) and pairwise comparisons (**B**)

The mean values of the CPS scores across all the 68 readers were derived for each assay and plotted across the samples (Fig. 2). In 50 cases, SP263 had the highest CPS score of the four antibodies, followed by 22C3 and E1L3N, whereas SP142 had a relatively lowest CPS score. Comparison of the four antibodies showed that 22C3 had good concordance with SP263, and the overall concordance rates of CPS, TCPS and ICPS were 0.896 (0.882–0.910), 0.884 (0.871–0.897) and 0.859 (0.843–0.875) respectively (Table 2).E1L3N and SP142 showed high concordance, and the overall concordance rates of CPS, TCPS and ICPS were 0.908 (0.866–0.919), 0.934 (0.923–0.945) and 0.859 (0.843–0.875) respectively (Supplementary Table 1). The concordance between 22C3 and SP142, 22C3 and E1L3N, SP263 and SP142, SP263 and E1L3N are relatively low (Supplementary Table 1).

**Table 2:** Concordance of PD-L1 Staining between the 22C3 and SP263

(N = 3400)			
Measurements	CPS	TCPS	ICPS
22C3 VS SP263			
CPs	3046 (89.6%)	3006 (88.4%)	2921 (85.9%)
DCPs	354 (10.4%)	394 (11.6%)	479 (14.1%)
Measures of agreement (95% CI)			
OPA	0.896 (0.882–0.910)	0.884 (0.871–0.897)	0.859 (0.843–0.875)
22C3 VS SP142			
CPs	2832 (83.3%)	2853 (83.9%)	2666 (78.4%)
DCPs	568 (16.7%)	547 (16.1%)	734 (21.6%)
Measures of agreement (95% CI)			
OPA	0.833 (0.813–0.853)	0.839 (0.824–0.853)	0.784 (0.762–0.807)
22C3 VS E1L3N			
CPs	2900 (85.3%)	2893 (85.1%)	2672 (78.6%)
DCPs	500 (14.7%)	507 (14.9%)	728 (21.4%)
Measures of agreement (95% CI)			
OPA	0.853 (0.840–0.867)	0.851 (0.836–0.865)	0.786 (0.764–0.809)
SP263 VS SP142			
CPs	2778 (81.7%)	2615 (76.9%)	2693 (79.2%)
DCPs	622 (18.3%)	785 (23.1%)	707 (20.8%)
Measures of agreement (95% CI)			
OPA	0.817 (0.794–0.840)	0.769 (0.748–0.790)	0.792 (0.772–0.812)
SP263 VS E1L3N			
CPs	2788 (82.0%)	2655 (78.1%)	2747 (80.8%)
DCPs	612 (18.0%)	745 (21.9%)	653 (19.2%)
Measures of agreement (95% CI)			
OPA	0.820 (0.801–0.838)	0.781 (0.762–0.800)	0.808 (0.790–0.862)
SP142 VS E1L3N			
CPs	3087 (90.8%)	3176 (93.4%)	2958 (87.0%)
DCPs	313 (9.2%)	224 (6.6%)	442 (13%)
Measures of agreement (95% CI)			
OPA	0.908 (0.896–0.919)	0.934 (0.923–0.945)	0.870 (0.856–0.884)

*N = C^2^_68_ (the number of comparison pairs of each case) × 50 (the number of cases)

*CI* confidence interval, *CP* concordant pair, *DCP* discordant CP, *NPA* negative percentage agreement, *OPA* overall percentage agreement, *PPA* positive percentage agreement

### Identification of cutoff values of CPS scores to improve agreement between 22C3 and the other three assays

**Fig. 3:**
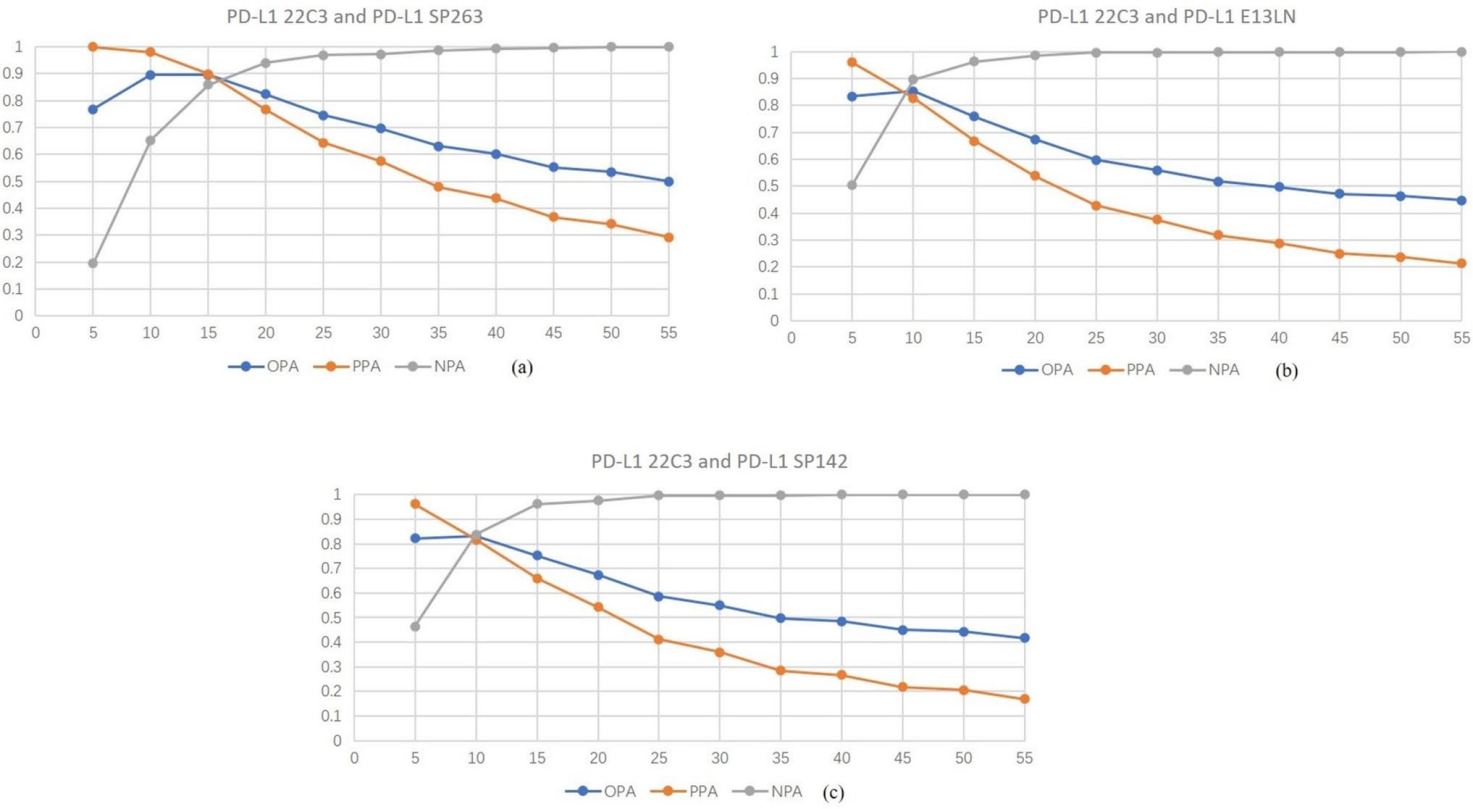
Taking PD-L1 22C3 CPS 10 as the positive threshold as the benchmark, when the positive threshold of the other three antibodies is changed, the overall concordance rate of 22C3 with the other three antibodies: **a** 22c3 and SP263, **b** 22c3 and E1L3N, and **c** between 22c3 and SP142

The FDA has approved PD-1/PD-L1 checkpoint inhibitors for locally advanced or metastatic esophageal cancer patients, and PD-L1 (22C3) CPS score ≥ 10 is used as a companion diagnosis for its second-line treatment. Therefore, in this study, we used 22C3 CPS ≥ 10 as the reference value, and by changing the positive thresholds of thethree antibodies SP263, SP142, and E1L3N, we sought the positive threshold when the overall agreement rate with 22C3 was the highest, which can reflect the substitution relationship between different antibodies to a certain extent. As shown in Fig. 3, SP142 and E1L3N have the highest overall agreement rate with 22C3 at a positive threshold of 10, with OPA of 0.82 and 0.86 respectively. When setting the positive threshold of SP263 at 10 or 15, the overall agreement rate with 22C3 is 0.9. However, at a positive threshold of 12, the overall agreement rate achieves its highest value, reaching an OPA of 0.92. And in the 4 cases of disagreement, 3 cases of 22C3 expression were negative, SP263 expression was positive, 1 case 22C3 expression was positive, SP263 expression was negative. SP263 did not cover all 22C3 positive cases.

## Discussion

China has a high incidence of esophageal cancer, characterized by poor prognosis and low 5-year survival rates. The National Medical Products Administration (NMPA) approved the Pembrolizumab for second-line treatment of PD-L1 (22C3) CPS ≥ 10 locally advanced or metastatic esophageal squamous carcinoma (ESCC), marking the initiation of immunotherapy for esophageal cancer. Accurate PD-L1 score evaluation is crucial. Despite numerous studies on PD-L1 in non-small cell lung cancer, especially those with limited pathologists resulting in high reproducibility, similar studies are lacking for esophageal cancer. In our study, we recruited 68 pathologists from 19 different medical centers to separately perform CPS interpretation scores for each of these four antibodies 22C3, SP263, SP142, and E1L3N, and to individually interpret the tumor cells and immune cells of the molecules in the formula. To the best of our knowledge, this is the first study to perform evaluations of the four PD-L1 antibodies in ESCC, with the largest number of pathologists, coming from multiple centers and possessing diverse clinical practice experience. Not only did we evaluate the concordance of PD-L1 interpretations in ESCC among pathologists, but also influencing factors were analyzed. We explored the impact of cell type on interobserver reproducibility by analyzing CPS, tumor cells, and immune cells concordance. Simultaneously, we assessed concordance among different antibodies, aiding clinicians and pathologists in choosing optimal PD-L1 antibodies and assays.

This study used routine esophageal squamous carcinomas samples usually encountered in actual clinical pathology work. All samples were scanned into digital slides for interpretation.Bethany Jill Williams’ research (Williams et al. 2019) confirmed that the assessment of immunohistochemistry (IHC) using scanned slides and mounted slides exhibits a high level of consistency. The Blue Print 2 study (Sound et al. 2018) validated a very strong correlation and consistency between the assessment of PD-L1 IHC results using scanned slides and mounted slides. Therefore, we adopted the method of scanning slices, achieving the goal of simultaneous and independent PD-L1 assessment by multiple individuals.The scores from 68 pathologists revealed that, except for SP263 CPS, which had a slightly higher concordance than TCPS (0.790 vs 0.762), 22C3, SP142, and E1L3N TCPS exhibited the best concordance, with OPA values of 0.80, 0.85, and 0.86, respectively. Our result is in harmony with the best concordance in the TPS assessment of non-small cell lung cancer obtained by several pathologists in Blueprint 2 (Sound et al. 2018). However, our study, despite unified training, saw lower concordance rates, likely due to the diverse field experiences of the numerous pathologists involved. In ICPS analysis, despite unified training, concordance was consistently low for all four antibodies, highlighting the challenge in evaluating immune cells, a challenge mirrored in esophageal squamous carcinoma and Blueprint 2. As positive immune cells influenced CPS interpretation, overall concordance was lower than TCPS and higher than ICPS, except for SP263.

In a multi-institutional NSCLC study (Rimm et al. 2017), pathologists exhibited excellent concordance in scoring tumor cells stained with any antibody but poor concordance for scoring immune cells. Our study also revealed a low overall concordance rate for ICPS, impacting PD-L1 CPS scores similarly to a urothelial carcinoma study (Hodgson et al. 2018), where immune cell staining reliability was lower compared to tumor cell staining (ICC 0.519–0.866). This underscores the need for pathologists interpreting PD-L1 to focus on accurate identification of immune cells (lymphocytes and macrophages), staining technique intensity, and sites. Further analysis indicated that cases near the threshold were more likely controversial, leading to reduced concordance. Among 50 cases, 22C3, SP263, SP142, and E1L3N had 33, 30, 34, and 33 discordant cases, respectively, with 29, 26, 28, and 31 having a score interval between 5 and 20. In contrast to some studies (Cooper et al. 2017; Chang et al. 2019; Paul et al. 2018) where pathologists had high concordance at 1% threshold and lower concordance at 50% and 25%, a threshold of 10 in our study seemed more subjective than thresholds of 1 and 50. Despite unified training, choosing cases near the threshold proved challenging, directly impacting patient treatment choices. Identifying specific threshold case characteristics, enhancing pathologist training around these cases, or leveraging new methods like artificial intelligence are crucial for improving reproducibility, accuracy, and providing precise guidance for clinical treatment.

In a previous study on head and neck squamous cell carcinoma, Hodgson et al. (2018) compared SP263 and 22C3 expression, in 27 surgically resected hypopharyngeal tumors and concluded that SP263 had a higher positivity rate and that there was good concordance between the two. With respect to lung cancer, both the Blue Print 2 and Munari et al. (2018) studies also found a significantly lower positive rate for 22C3 compared with SP263 and showed significant differences in the selection of beneficiary patients at clinically relevant thresholds. In our esophageal squamous cell carcinoma study, SP263 exhibited the highest sensitivity, followed by 22C3, E1L3N, and SP142. Overall CPS concordance rates were slightly higher than 22C3, SP142, and E1L3N. The best concordance was between SP263 and 22C3 (OPA 0.896), consistent with NSCLC findings (Buttner and Gosney. 2017). In the analysis of SP263 versus 22C3 expression in 108 HNSCC biopsy samples, Ratcliff et al. (2016) raised the possibility of using the two interchangeably for analysis. Munari et al. mentioned that SP263 versus 22C3 expression showed significant differences in the selection of beneficiary patients at clinically relevant thresholds. Our results revealed three discordant cases negative for 22C3 but positive for SP263 and one case positive for 22C3 but negative for SP263, indicating good concordance but varied conclusions on interchangeability. Further investigation is needed to assess the impact on PD-L1-positive cases and clinical response rates. Due to pathologist workload and interpretation time, our study had limitations with a sample size of 50 cases and inclusion of surgical specimens.

In summary, this study is the first multi-center concordance study of four antibodies, 22C3, SP263, SP142, and E1L3N, in ESCC. SP263 expression had the highest scores. 22C3 and SP263 had the best concordance; however, the results of this study do not support the interchangeability of SP263 and 22C3 standardized analysis when used to determine PD-L1 expression but has certain reference value of their clinical use of ESCC for assessment of PD-L1 expression.

### Supplementary Information

Below is the link to the electronic supplementary material.

Supplementary file1 (JPG 363 KB)

Supplementary file2 (PNG 1461 KB)

Supplementary file3 (DOCX 18 KB)

## Data Availability

The raw/processed data required to reproduce these findings cannot be shared at this time as the data also forms part of an ongoing study.
