# Supplementary figures and images for: Concordance of assessments of four PD-L1 immunohistochemical assays in esophageal squamous cell carcinoma (ESCC)
Source: J Cancer Res Clin Oncol. 2024 Jan 28;150(2):43. doi: 10.1007/s00432-023-05595-0 (PMC10821831; doi:10.1007/s00432-023-05595-0)

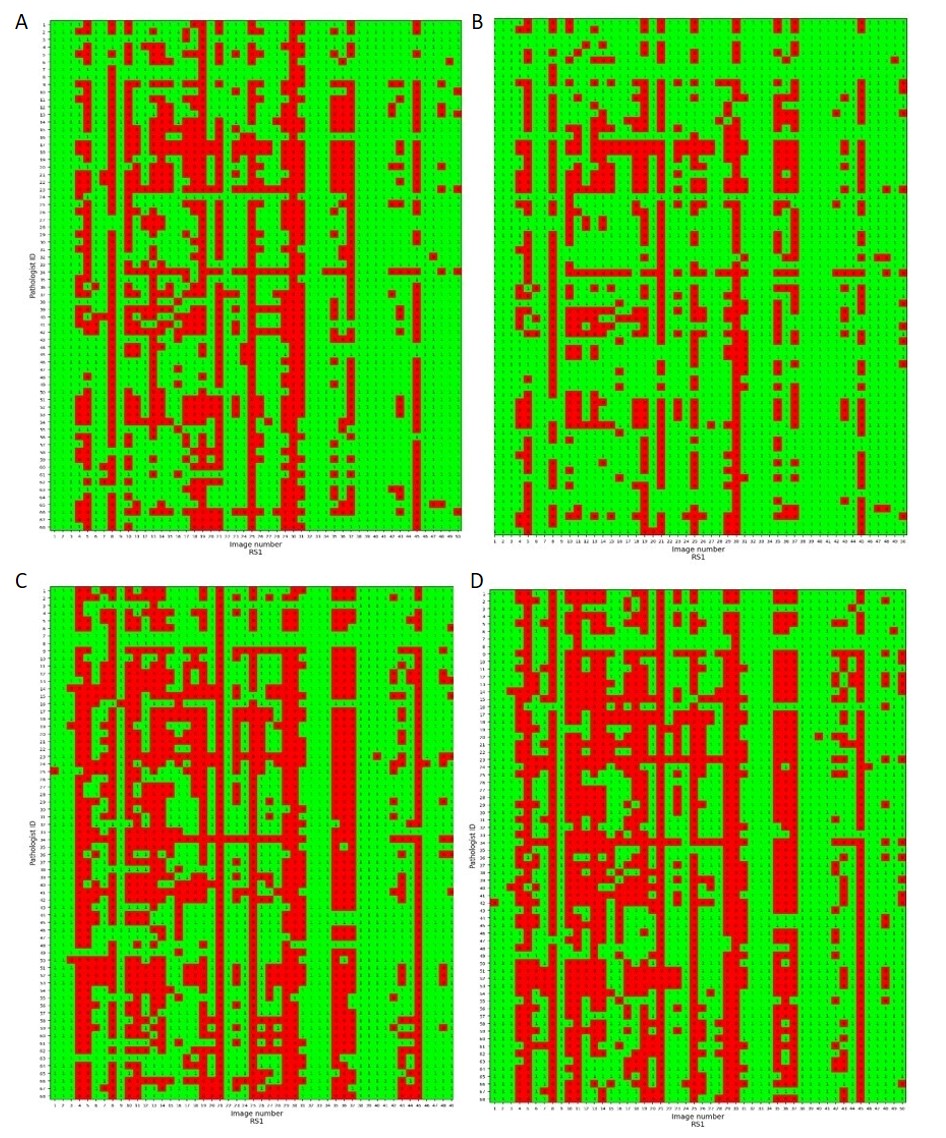

Supplement: Supplementary file 1 — Supplementary file1 (JPG 363 KB) [file 432_2023_5595_MOESM1_ESM.jpg]

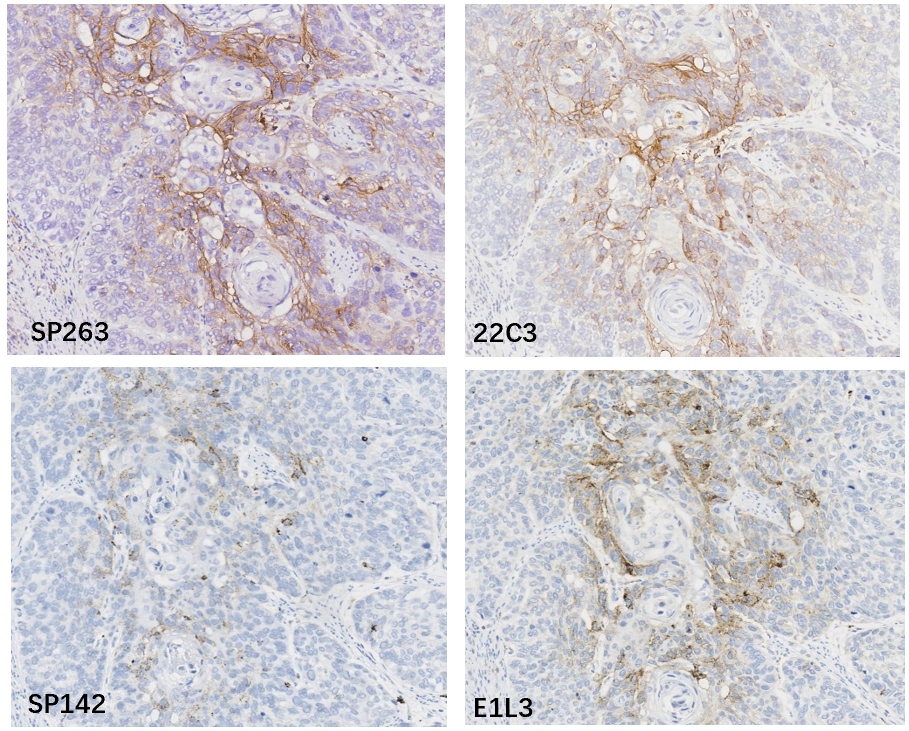

Supplement: Supplementary file 2 — Supplementary file2 (PNG 1461 KB) [file 432_2023_5595_MOESM2_ESM.png]
