# Supplementary material for: Concordance of assessments of four PD-L1 immunohistochemical assays in esophageal squamous cell carcinoma (ESCC)
Source: J Cancer Res Clin Oncol. 2024 Jan 28;150(2):43. doi: 10.1007/s00432-023-05595-0 (PMC10821831; doi:10.1007/s00432-023-05595-0)
Supplement: Supplementary file 3 — Supplementary file3 (DOCX 18 KB) [file 432_2023_5595_MOESM3_ESM.docx]

Supplementary table 1 Consistency of PD-L1 Staining between the PD-L1 assays

| 22C3 VS SP142 | | | |
| --- | --- | --- | --- |
| Measurements | CPS | TCPS | ICPS |
| CPs | 2832(83.3%) | 2853(83.9%) | 2666(78.4%) |
| DCPs | 568(16.7%) | 547(16.1%) | 734(21.6%) |
| Measures of agreement (95% CI) | | | |
| OPA | 0.833(0.813-0.853) | 0.839(0.824-0.853) | 0.784(0.762-0.807) |
| 22C3 VS E1L3 | | | |
| Measurements | CPS | TCPS | ICPS |
| CPs | 2900(85.3%) | 2893(85.1%) | 2672(78.6%) |
| DCPs | 500(14.7%) | 507(14.9%) | 728(21.4%) |
| Measures of agreement (95% CI) | | | |
| OPA | 0.853(0.840-0.867) | 0.851(0.836-0.865) | 0.786(0.764-0.809) |
| SP263 VS SP142 | | | |
| Measurements | CPS | TCPS | ICPS |
| CPs | 2778(81.7%) | 2615(76.9%) | 2693(79.2%) |
| DCPs | 622(18.3%) | 785(23.1%) | 707(20.8%) |
| Measures of agreement (95% CI) | | | |
| OPA | 0.817(0.794-0.840) | 0.769(0.748-0.790) | 0.792(0.772-0.812) |
| SP263 VS E1L3 | | | |
| Measurements | CPS | TCPS | ICPS |
| CPs | 2788(82.0%) | 2655(78.1%) | 2747(80.8%) |
| DCPs | 612(18.0%) | 745 (21.9%) | 653(19.2%) |
| Measures of agreement (95% CI) | | | |
| OPA | 0.820(0.801-0.838) | 0.781(0.762-0.800) | 0.808(0.790-0.862) |
| SP142 VS E1L3 | | | |
| Measurements | CPS | TCPS | ICPS |
| CPs | 3087(90.8%) | 3176(93.4%) | 2958(87.0%) |
| DCPs | 313(9.2%) | 224(6.6%) | 442(13%) |
| Measures of agreement (95% CI) | | | |
| OPA | 0.908(0.896-0.919) | 0.934(0.923-0.945) | 0.870(0.856-0.884) |

N=C**^2^_68_**(the number of comparison pairs of each case) ×50 (the number of cases).

CI, confidence interval; CP, concordant pair; DCP, discordant CP; NPA, negative percentage agreement; OPA, overall percentage agreement; PPA, positive percentage agreement.

Supplementary table 2 Clinicopathological Characteristics of patients

| Characteristic | | Case（N＝50） |
| --- | --- | --- |
| Age | ≤50years | 23（46.0%） |
|  | ＞50years | 27（54.0%） |
| Histological grade | Ⅰ | 4（8.0%） |
|  | Ⅱ | 40（80.0%） |
|  | Ⅲ | 6（12.0%） |
| Tumor size | ＜2cm | 8（16.0%） |
|  | 2cm-3cm | 35（70.0%） |
|  | ＞3cm | 7（14.0%） |
| Lymph node metastasis | None | 10（20.0%） |
|  | 1-2 | 25（50.0%） |
|  | ≥3 | 15（30.0%） |
| TNM stage | Ⅱ | 25（50.0%） |
|  | Ⅲ | 23（46.0%） |
|  | Ⅳ | 2（4.0%） |
